# Synthesis of New Cyclodextrin-Based Adsorbents to Remove Direct Red 83:1

**DOI:** 10.3390/polym12091880

**Published:** 2020-08-20

**Authors:** José A. Pellicer, María Isabel Rodríguez-López, María Isabel Fortea, Vicente M. Gómez-López, David Auñón, Estrella Núñez-Delicado, José A. Gabaldón

**Affiliations:** Departamento de Ciencias de la Salud. Universidad Católica San Antonio de Murcia (UCAM), Avenida de los Jerónimos 135, Guadalupe, 30107 Murcia, Spain; japellicer@ucam.edu (J.A.P.); mirodriguez@ucam.edu (M.I.R.-L.); mifortea@ucam.edu (M.I.F.); vmgomez@ucam.edu (V.M.G.-L.); daunon@alu.ucam.edu (D.A.); enunez@ucam.edu (E.N.-D.)

**Keywords:** cyclodextrins, adsorption, polymerization, wastewater treatment, pulsed light

## Abstract

Two cyclodextrins (CDs), γ– and hydroxypropyl (HP)–γ–CDs were used to synthesize new adsorbents by using epichlorohydrin (EPI) as cross-linking agent in order to remove Direct Red 83:1 (DR) from water. Both polymers were characterized in terms of Fourier spectroscopy, nuclear magnetic resonance, particle size distribution and thermogravimetric analysis. Experimental data for both polymers were well fitted to the pseudo-second order and intraparticle diffusion model, indicating that in the adsorption both chemical and physical interactions are essential in the removal of DR. Three different isotherm models were analyzed, concluding that γ–CDs–EPI followed the Temkin isotherm and HP–γ–CDs-EPI the Freundlich isotherm, these results suggested that the adsorption was happening onto heterogeneous surfaces. The results of the Gibbs free energy showed that the adsorption was spontaneous at room temperature. In order to eliminate the remaining dye after the polymer treatment, and advanced oxidation process (AOP) was considered, achieving more than 90% of removal combining both mechanisms.

## 1. Introduction

There is a huge concern related to the release of emerging contaminants in the environment, this issue is considered nowadays as a worldwide risk. Emerging pollutants encompass a wide range of man-made chemicals (such as pesticides, cosmetics, personal and household care products, pharmaceuticals, textile dyes, etc.), which are in use worldwide and which are indispensable for modern society. Human activities have led to the contamination of water resources with micropollutants. Such agents have generated renewed awareness due to their potential pathogenicity.

Textile industries use a wide range of direct dyes to colour their goods. Apart from that, the consumption of water gives rise to an average of 200 L/kg of fibre, producing a large volume of wastewater [1]. The most common dyes used in the textile industry are sulfur, indigoid, anthraquinone, triphenylmethyl and azo derivatives, being azo dyes the most employed because of their high solubility in aqueous solution [2]. The discharge of dyes into water effluents is extremely dangerous due to the undesirable characteristics that will transfer to the ambient [3]. Therefore, due to the high restrictions related to the discharge of dyes in the effluents, it is mandatory to treat water before discharging it into the environment [4].

Conventional methods to remove dyes from wastewater are not effective due to their resistant to aerobic biodegradation, heat, light and oxidizing agents. One remarkable alternative is the use of adsorption techniques for the removal of dyes. Adsorption processes are considered the most useful method because the advantages clearly outweigh the potential disadvantages [5]. In order to perform this process, many different adsorbents are being used, such as: activated carbon [6], chitosan [7], magnetic chitosan resins [8], magnetic thiourea-formaldehyde resins [9], silica [10], graphene [11], zeolite [12], Dowex resin [13], alginate beads [14], montmorillonite clay [15] and cyclodextrins [16,17].

Cyclodextrins (CDs) are produced from starch. CDs are cage molecules, so the core of the structure is composed of a stable hydrophobic cavity that can entrap or encapsulate other molecules [18]. Depending on the number of carbons, three different native CDs are available (α-CDs, β-CDs and γ–CDs). To increase the aqueous solubility of the CDs, some chemical modifications have been carried out, being the addition of hydroxyl groups, the most common [19].

CDs have the ability to form polymeric structures due to the interaction with crosslinking agents. This stems from the fact that the presence of –OH groups on the CDs can lead to the formation of stable bonds with different crosslinking compounds then giving rise to water-insoluble structures [20]. One of the most typical methods to produce insoluble CDs polymer involved the utilization of epichlorohydrin (EPI). This agent contains an epoxide and a chloroalkyl reactive group that are necessary to interact with the CDs and thanks to this interaction, the polymeric structure will be obtained [21].

Given the fact that the cyclodextrin adsorption process is unable to eliminate the totality of the contaminant, an advanced oxidation process can be used to further decrease the amount of dye remaining in the solution. This will decrease the amount of dye that would be eventually transferred to the environment. A H_2_O_2_/pulsed light advanced oxidation process (AOP) might be a suitable solution. Pulsed light (PL) is considered an emerging technology within the field of food technology but also with potential to be implemented in non-food-related applications because it generates a high flux of photons, which enables it to render photolytic effects in relatively short times. It is based in the repetitive application of high-power short-time pulses of wide-spectrum incoherent light, the emission of whose spectrum includes ultraviolet (UV) light [22]. Its use as light source in the frame of an advanced oxidation process for textile dye degradation has been recently described [23].

The main aim of this work was the preparation of γ– and HP–γ–CDs polymerized with EPI for the first time to remove Direct Red 83:1 (DR) from wastewater. Both adsorbents were characterized in terms of nuclear magnetic resonance (NMR), Fourier transform infrared spectroscopy (FTIR), thermogravimetric analysis (TGA) and particle size distribution. To understand the adsorption process, the experimental data were fitted to different kinetics and isotherms. Finally, the efficacy of a H_2_O_2_/PL AOP was tested to degrade the unadsorbed amount of dye in order to generate cleaner water.

## 2. Materials and Methods

### 2.1. Chemicals

CDs used in the experiments were obtained from AraChem (Tilburg, The Netherlands), sodium borohydride (NaBH_4_), sodium hydroxide (NaOH), epichlorohydrin (EPI) and acetone were from Sigma-Aldrich (Madrid, Spain). The dye (Direct Red 83:1) was kindly provided by Colorprint (Alcoy, Spain).

### 2.2. Polymer Synthesis

**Figure 1:**
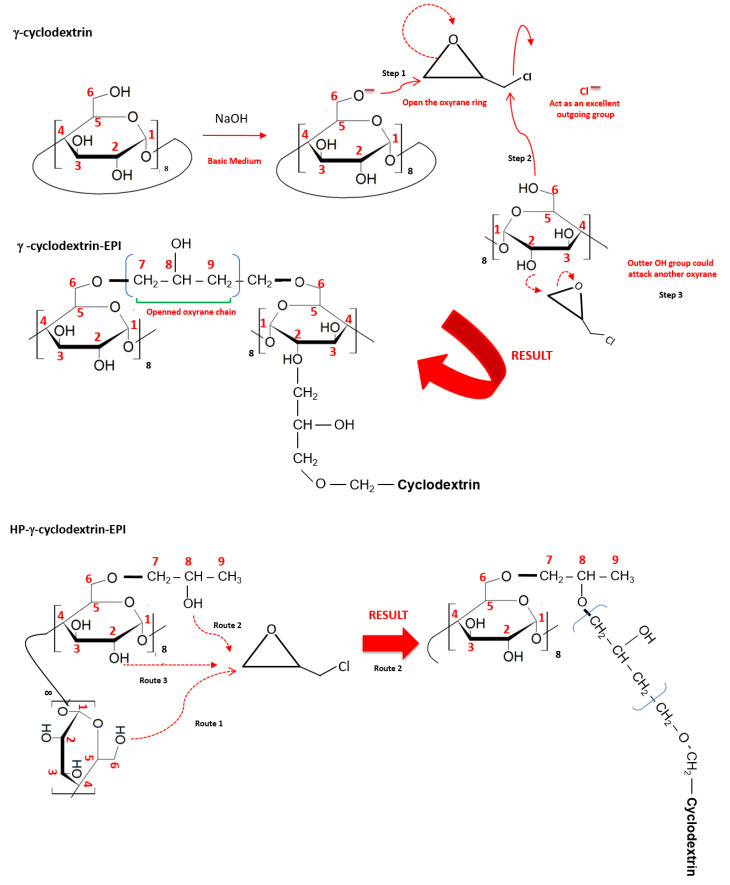
Scheme of reactions taking place between cyclodextrins (CDs) and epichlorohydrin (EPI).

The synthesis of insoluble polymers containing γ– and HP–γ–CDs crosslinked with EPI was carried out by using the standardized protocol employed in our previous articles [16,17]. Figure 1 describes the chemical reactions that lead to the synthesis of both polymeric structures.

### 2.3. Adsorption Experiments

The experiments were conducted at room temperature using a set of Direct Red (DR) concentrations (from 25 to 300 mg/L). Flasks containing 50 mL of each concentration were mixed with 1 g of adsorbent. This mixture was agitated at 500 rpm and at specific intervals, aliquots were extracted from the samples in order to analyse the remaining dye in the liquid solution. Prior to measure the absorbance at 526 nm, the samples were centrifuged to discard impurities. 

The dye entrapped on the polymeric adsorbents was analysed by using the following Equation (1) [24]:

(1) $$q_{e}=\frac{V(C_{o}-C_{e})}{m}$$

where *C*_o_ is the initial dye concentration (mg/L), *C*_e_ the liquid phase dye concentration at equilibrium (mg/L), *V* the volume of dye used (L) and *m* is the mass of polymer utilized (g). Three replicates were performed and plotted.

### 2.4. Polymer Characterization

Fourier transform infrared spectroscopy (FTIR) spectra, nuclear magnetic resonance (NMR) spectra, particle size distribution, thermogravimetric analysis (TGA) and surface morphologies were carried out in order to characterize the polymer as reported before [25].

FTIR spectra of the samples were collected in a VARIAN FT-IR 670 in the range 4000–400 cm^−1^ in attenuated total reflection (ATR) mode with a spectral resolution of 0.1 cm^−1^.

Nuclear magnetic resonance (NMR) analysis was performed in a Bruker Advance AV300 MHz spectrometer. Briefly 40 mg of the sample were dissolved or suspended in 1ml deuterium oxide (D_2_O), filtered through 0.45 µm and analyzed for ^1^H NMR spectra in the chemical shift range of 14 to −1 ppm.

Particle size distribution was studied by laser diffraction using acetone as solvent in a Mastersizer 3000E (Malvern Panalytical).

The porosity and apparent density of the polymers was calculated using the following equations:

(2) Porosity (%)=Vt−VaVt×100= Vt−MaρVt×100

(3) $$\mathrm{Density}\;\left(\rho \right)=\frac{V_{t}}{M_{a}}$$

where *V*_t_ (cm^3^) is the total volume of polymers, *V*_a_ (cm^3^) is the actual volume of the material, *M*_a_ (g) is the mass of the polymers and ρ (g/cm^3^) is the density of the material. The experiments were repeated three times.

The swelling capacity of the polymers was measured by using a gravimetric method: 1 g of dry samples was immersed in 200 mL of distilled water at room temperature for 3 h to reach swelling equilibrium. After this time, both polymers were filtered to remove unabsorbed water. The swelling equilibrium (*Q*_eq_ g/g) was determined according to the following equation:

(4) $$Q_{\mathrm{eq}}=\frac{W_{s}-W_{d}}{W_{d}}\;$$

where *W*_d_ is the mass of dried polymers (g) and *W*_s_ is the mass of swollen polymers (g). The experiments were repeated three times [26].

For thermogravimetric analysis (TGA) between 1–3 mg of each sample in aluminium pans were introduced in a Discovery TGA55 (TA Instruments) thermogravimetric instrument. After stabilization, the samples were subjected to heat scanning from 25 to 350 °C at 10 °C·min^−1^ under nitrogen atmosphere and the loss of weight recorded and compared.

### 2.5. Advanced Oxidation Process (AOP)

The highest dye concentration remaining after the adsorption processes was 80–90 mg/L, which corresponded to the tests with initial concentration of 300 mg/L for both dyes. In order to degrade that concentration of dye, 20 mL of a mixture of dye and hydrogen peroxide at final concentrations of 86 mg/L (87 µM) and 1480 mg/L (43 mM), respectively, was prepared in a Petri dish and subjected to pulsed light treatment. A high ratio hydrogen peroxide/dye was used in order to avoid make the first the limiting reagent of the reaction.

The Petri dish without cover was placed in a pulsed light device (XeMaticA-Basic-1L, Steribeam, Germany). The system was operated at 2.5 kV and produced a light fluence of 2.14 J/cm^2^ at the surface of the liquid. Under these conditions, the emission of the lamp has a spectrum similar to the reported Cudemos et al., [27]. Increasing fluences up to 193 J/cm^2^ were reached by applying multiple pulses. Tests were carried on in triplicate.

Pseudo-first order degradation constants (k, cm^2^/J) were calculated as function of fluence (*H*_o_, J/cm^2^) according to the following equation:

(5) $$\ln\frac{C}{C_{o}}=\;-kH_{o}$$

where *C* is the concentration of dye at fluence *H*_o_ and *C*_o_ is the initial dye concentration.

## 3. Results and Discussion

### 3.1. Polymer Characterization

**Figure 2:**
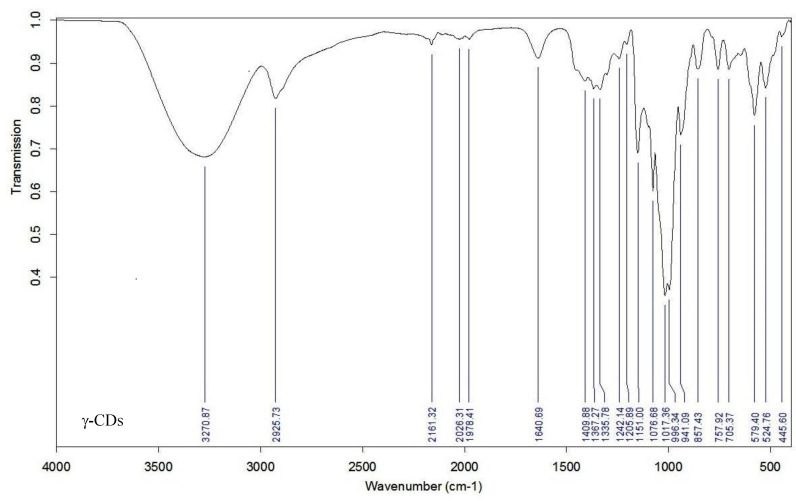

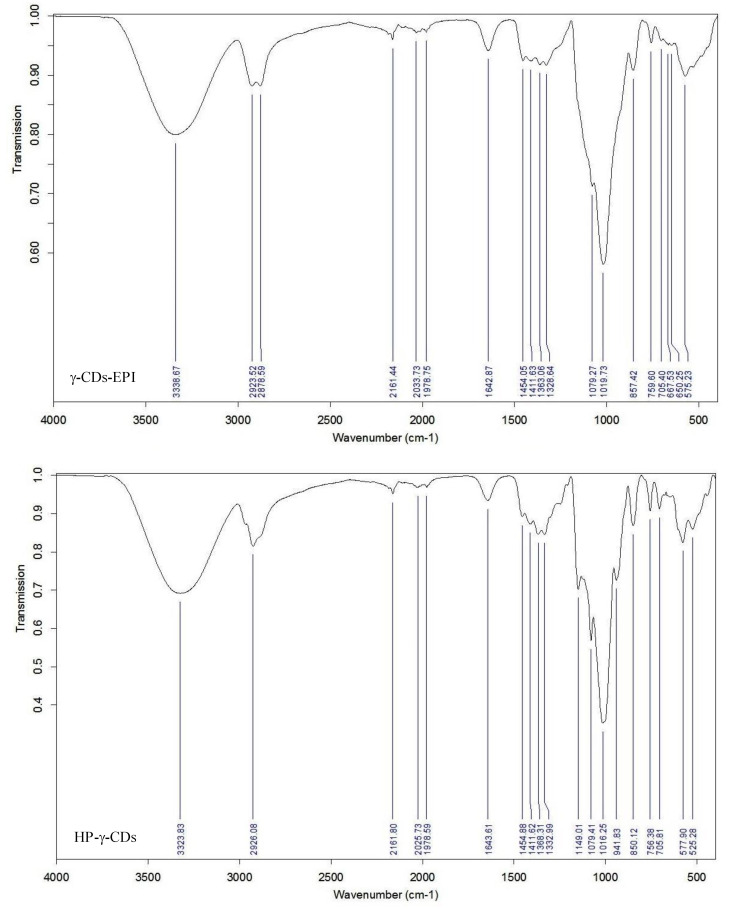

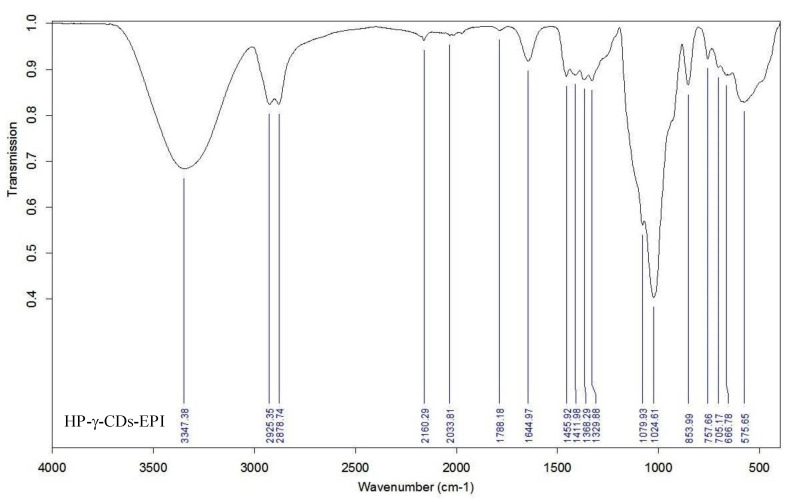
Fourier transform infrared (FTIR) spectra for the native and modified CDs and the crosslinked polymers.

In the infrared (IR) spectra of γ–CD, HP–γ–CD and their corresponding polymers (Figure 2) a wide band was observed between 3400–3200 cm^−1^. This band is attributed to the stretching vibrations of either the O–H bonds of the primary and secondary –OH groups of the glucopyranose units and the isopropyl moieties in the parent cyclodextrins, and to the remaining not linked C–OH groups in the polymers. An absorption band is also observed, belonging to the stretching vibrations of the C–H bonds in the CH and CH_2_ groups with a maximum around 2920 cm^−1^. In polymeric compounds an additional band appears at ca. 2880 cm^−1^ responding to the same sort of vibration from the new carbon backbone bonded to the cyclodextrin rings. 

Regarding the bands around the 1640 cm^−1^ region, already present in the parent compounds and maintained in the polymers derived, can be assigned to the bending deformation of the water molecules H–O–H attached to the cyclodextrin rings. The absorption bands ranging from 1400 to 400 cm^−1^ (fingerprint region) are grouped in three main regions and can be attributed to deformation vibrations C–O–H at 1450–1200 cm^−1^, stretching vibrations C–O–C at 1260–970 cm^−1^ and other minor unspecific C–H deformation bands at 800–400 cm^−1^ belonging to the deformation vibrations of the C–H bonds and the vibrations of the glucopyranose cycle.

The ^1^H NMR spectrum (Figure S1, Supplementary Material) of the two polymeric structures and their parent cyclodextrins was performed in D_2_O to record protons bonded other than OH groups. Compared to the cyclodextrins the appearance of two novel signals at medium and low chemical shift together with their high intensity related to the signal of the parent compounds suggests a high level of crosslinking. Nevertheless, no additional information could be drawn from the spectrum of these polymers due to their low solubility and additional experiments will be undertaken to unravel the internal organization of the polymers.

**Figure 3:**
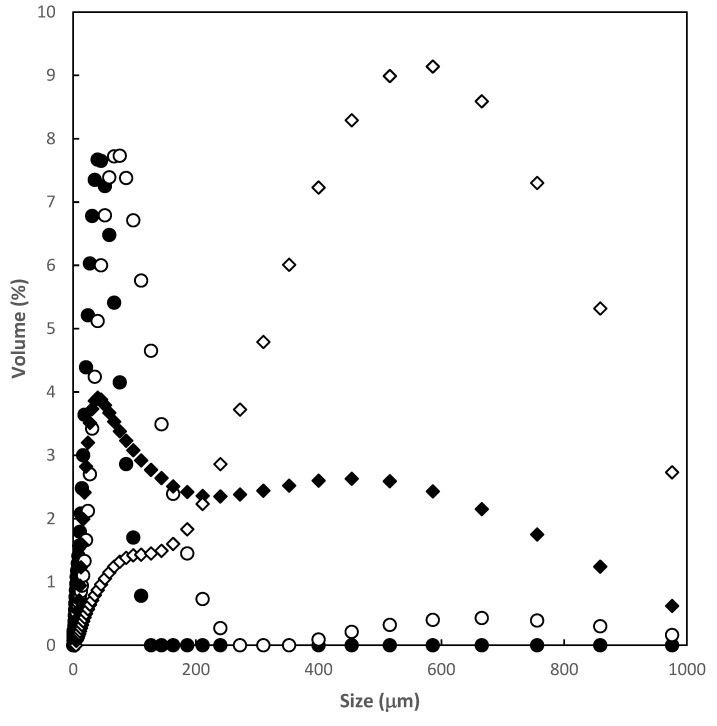
Particle size distribution (γ–CDs (•), HP–γ–CDs (○), γ–CDs-EPI (♦), HP–γ–CDs-EPI (⟡)).

Particle size distribution (Figure 3) was analysed in terms of the volumetric size (D[4:3]). Comparing both adsorbents, the result was higher for HP–γ–CDs–EPI (555 μm) than γ–CDs–EPI (54 μm). Span values were calculated by using the following Equation (6):

(6) $$Span=\;\frac{D_{90}-D_{10}}{D_{50}}$$

The results obtained were 1.8 for γ–CDs, 1.8 for HP–γ–CDs, 6.7 for γ–polymer and 1.6 for HP–γ–polymer. In this case, lower values are in accordance with more homogeneous particle distribution.

The characterization of both adsorbents involved the measure of the swelling capacity, porosity, density and the particle size distribution. The results obtained could be observed in Table 1. According to the results, both CD polymers showed similar properties in terms of swelling capacity, porosity and density. The most remarkable differences are related to the size of the particles and the homogeneity of them as explained previously.

**Figure 4:**
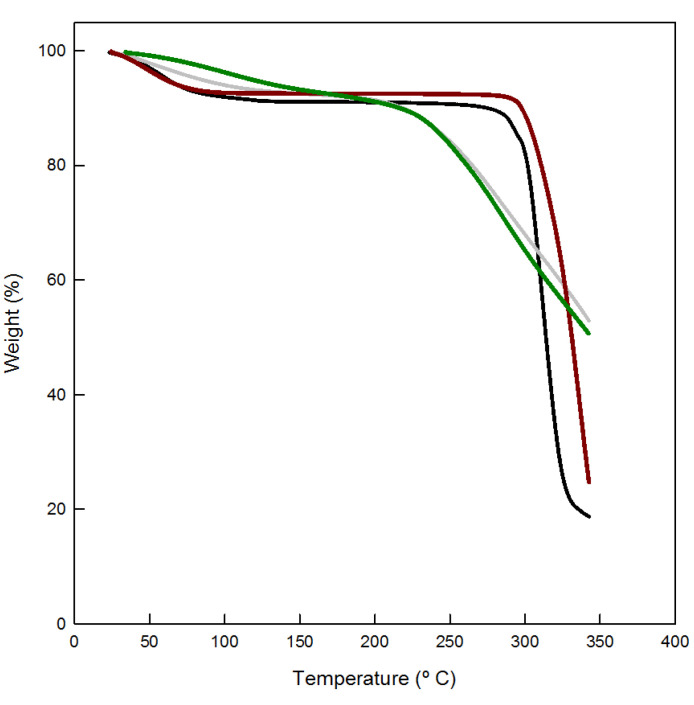
Thermograms for the different samples analyzed (black: γ–CDs, red: HP–γ–CDs, grey: γ–CDs–EPI, green: HP–γ–CDs–EPI).

It could be observed in all the thermograms (Figure 4) a first weight loss around 100 °C that can be explained by removal and evaporation of surface-adsorbed water molecules. In the case of polymeric structures this loss is observed at higher temperatures, probably due to an increase in the surface area that holds the molecules more efficiently. The second loss of weigh in the curves corresponds to the thermal decomposition of cyclodextrin oligosaccharides in the case of the parent compounds. In the EPI-crosslinked compounds the second process normally starts at lower temperatures with a gradually degradation of the alkyl backbones followed by the subsequent decomposition of the cyclodextrin structures.

### 3.2. Effect of Contact Time

The first step in the uptake of dyes from water is to determine the best adsorption conditions for the adsorbents. In order to obtain this information, it is essential to evaluate different factors, such as: adsorbent dosage, agitation speed or pH as the most important conditions. The main aim of these experiments is to measure the capability of the polymers synthesized to remove direct dyes from water by using the optimal adsorption conditions.

According to the results obtained, the following conditions were fixed to perform the adsorption experiments: 1 g of polymer, 500 rpm and pH 7. After evaluating the best adsorption conditions, the next step is to analyse the effect of contact time between the adsorbents and DR by using eight different concentrations.

**Figure 5:**
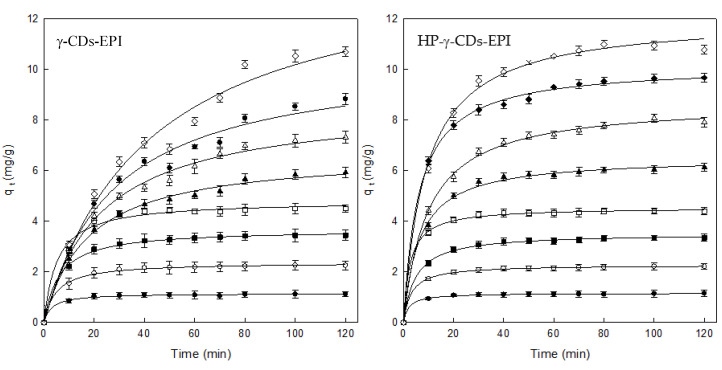
Contact time analysis between CD polymers and different concentrations of Direct Red (25 mg/L (●), 50 mg/L (○), 75 mg/L (■), 100 mg/L (□), 150 mg/L (▲), 200 mg/L (Δ), 250 mg/L (♦) and 300 mg/L (⟡)). Experimental conditions: adsorbent = 1g, pH = 7, speed = 500 rpm, contact time = 120 min, dye concentration = 25–300 mg/L.

The results of contact time for γ– and HP–γ–CDs–EPI could be seen in Figure 5. Analysing the results for γ–CDs–EPI, increasing the concentration of Direct Red gave rise to increasing q_t_ values for the whole range of concentration used. However, from 25 to 150 mg/L, the adsorption was very fast, reaching adsorption equilibrium after 40 min. In the case of the highest dye concentrations, the trend was clearly different, the equilibrium time increased from 40 to 80 min, this is due to the high capability of this adsorbent to entrap more dye molecules at high concentrations of DR.

For HP–γ–CDs–EPI the results were similar for the different concentrations, the adsorption was rapid, independently of dye concentration, according to Figure 4, the equilibrium time was reached after 30–40 min of adsorption.

### 3.3. Adsorption Kinetics

Adsorption kinetics were determined by adjusting the experimental data to three different models (pseudo-first (PFOM), pseudo-second (PSOM) and intraparticle diffusion (IDM) models, respectively). The linearized equations for these models are listed as follows [28,29,30]:

(7) $$\log\left(q_{e}-q_{t}\right)=\log q_{e}\;-\frac{k_{1}}{2.303}t$$

(8) $$\frac{t}{q_{t}}=\;\frac{1}{{k_{2}q_{e}}^{2}}+\;\frac{1}{q_{e}}t$$

(9) $$q_{t}=k_{i}\sqrt{t}+\;C\;$$

*q*_e_ and *q*_t_ are the quantity of dye adsorbed (mg/g), k_1_ (min^−1^) is the constant related to the pseudo-first model, k_2_ (g/mg min) is the constant related to the pseudo-second model, k_i_ (mg/g min ½) is the constant related to the intraparticle diffusion model, t is the time and C is the intercept (mg/g).

**Figure 6:**
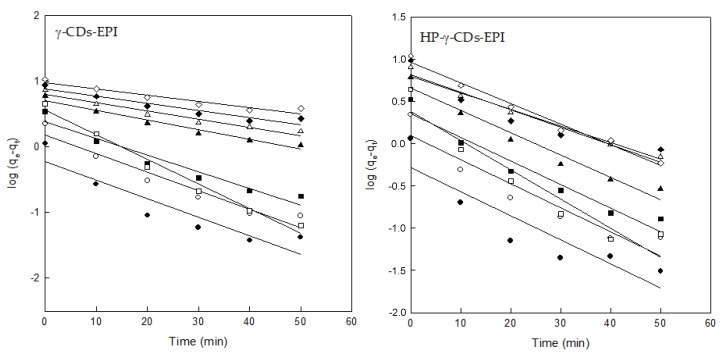
Pseudo-first order model analysis for both adsorbents and different DR concentrations (25 mg/L (●), 50 mg/L (○), 75 mg/L (■), 100 mg/L (□), 150 mg/L (▲), 200 mg/L (Δ), 250 mg/L (♦) and 300 mg/L (⟡)). Experimental conditions: adsorbent = 1g, pH = 7, speed = 500 rpm, contact time = 120 min, dye concentration = 25–300 mg/L.

The results observed for the adjustment to the PFOM for both adsorbents could be seen in Figure 6 and Table 2. With the objective to obtain the best representation possible, half of the values of contact time were not taken into account, so for the PFOM plot only the first 50 min of contact were considered. Using the whole range of measure (120 min), the straight lines obtained for each concentration showed a very poor determination coefficient (R^2^). In the case of 50 min of contact time, the experimental values obtained (Table 2) were similar in some cases to the calculated values using the PFOM. The R^2^ values ranged from 0.8 to 0.98, showing a high deviation between values. When this trend occurs, it is more likely that the adsorption of DR on both adsorbents might take place through the PSOM.

**Figure 7:**
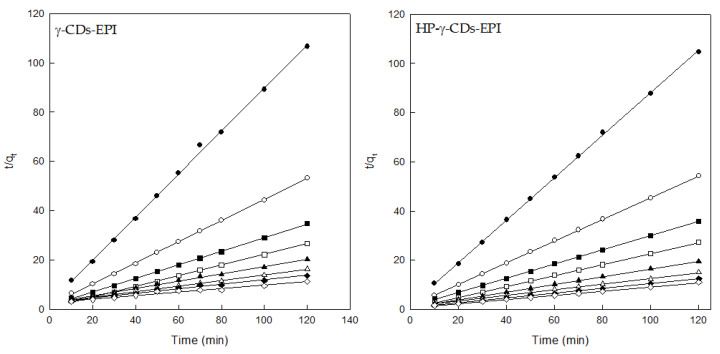
Pseudo-second order model analysis for both adsorbents and different DR concentrations (25 mg/L (●), 50 mg/L (○), 75 mg/L (■), 100 mg/L (□), 150 mg/L (▲), 200 mg/L (Δ), 250 mg/L (♦) and 300 mg/L (⟡)). Experimental conditions: adsorbent = 1g, pH = 7, speed = 500 rpm, contact time = 120 min, dye concentration = 25–300 mg/L.

The results for the PSOM adjustment demonstrated the perfect fit between the experimental data and this model. The determination coefficients confirmed these results for both adsorbents (Figure 7, Table 2). Due to these values, it is possible to confirm that chemical forces were playing an essential role in the adsorption of DR. The comparison of these results with previously published papers showed that similar kinetics were obtained using different dyes and adsorbents such as the removal of methylene blue on fly ash [31], the removal of acidic dyes on silica [32], the adsorption of DR on CDs [16,17] or the elimination of Direct Blue on chitosan [7].

**Figure 8:**
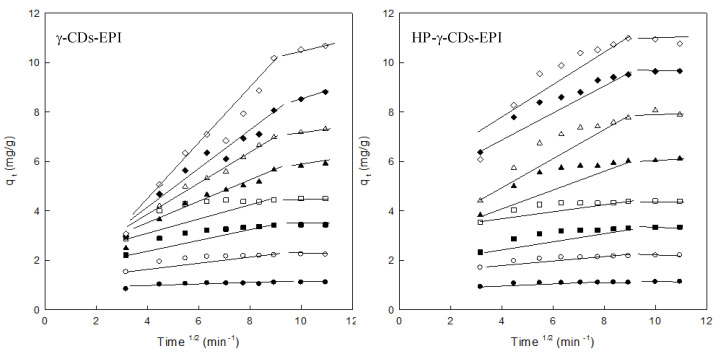
Intraparticle diffusion model analysis for both adsorbents and different DR concentrations (25 mg/L (●), 50 mg/L (○), 75 mg/L (■), 100 mg/L (□), 150 mg/L (▲), 200 mg/L (Δ), 250 mg/L (♦) and 300 mg/L (⟡)). Experimental conditions: adsorbent = 1g, pH = 7, speed = 500 rpm, contact time = 120 min, dye concentration = 25–300 mg/L.

To analyse the effect of the intraparticle diffusion on the adsorption of DR is necessary to consider the presence of different steps in this plot. Two different straight lines indicated us that two different forces are controlling the adsorption, in our case both chemical and intraparticle diffusion were playing a key role in this adsorption [33]. The first part represents the chemical adsorption and the second one the intraparticle diffusion. According to our results (Figure 8 and Table 2), both adsorbents showed this multi-step adsorption process. The first part of the plot is a straight line, indicating the surface adsorption for both polymers, the second part of the representation is a flat line, especially relevant at high DR concentrations, indicating that the IDM is important in the adsorption using cyclodextrin polymers. In this adsorption both chemical and intraparticle interactions are involved in the removal of this azo dye from water. Due to the presence of two different steps in the IDM it was not possible to obtain high R^2^ values. This values ranged from 0.575 to 0.960 for γ–CDs–EPI and from 0.649 to 0.865 in the case of HP–γ–CDs–EPI.

### 3.4. Adsorption Equilibrium

The analysis of the equilibrium adsorption state is very important as it allows understanding the adsorption mechanism. The experimental data were fitted using Freundlich, Langmuir and Temkin isotherm models that are the most common mechanisms to explain the aqueous phase adsorption and are expressed by using Equations (10)–(12) [34,35,36]:

(10) $$\ln q_{e}=\ln K_{F}\;+\;\frac{1}{n_{F}}\ln C_{e}$$

(11) $$\frac{C_{e}}{q_{e}}=\frac{1}{K_{L}}\;+\;\frac{a_{L}}{K_{L}}C_{e}\;$$

(12) $$q_{e}=\frac{RT}{b_{T}}\ln a_{T}\;+\;\frac{RT}{b_{T}}\ln C_{e}$$

*K*_F_ is the constant associated to the Freundlich isotherm (mg/g) (L/mg ^1/*n*^), *n*_F_ is the heterogeneity factor, *K*_L_ (L/g) and *a*_L_ (L/mg) are the constants related to the Langmuir model, *q*_max_ is defined by *K*_L_/*a*_L_. *b*_T_ is the constant related to the adsorption heat (kJ/mol), *a*_T_ is the constant related to the Temkin isotherm (L/g), *R* is the universal constant (8.314 J/mol K) and *T* is the temperature (K).

**Figure 9:**
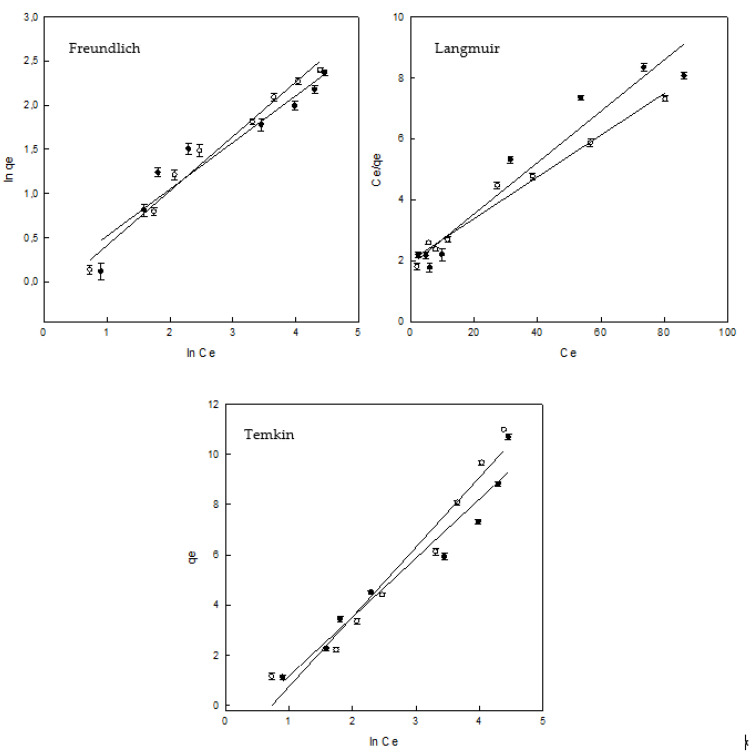
Freundlich, Langmuir and Temkin isotherms (γ–CDs–EPI (•), HP–γ–CDs–EPI (○)).

The Freundlich isotherm model presented the best fit for HP–γ–CDs–EPI, taking into account the R^2^ value (0.982). This means that heterogeneous surfaces are playing a key role in the adsorption of DR (Figure 9 and Table 3). The most useful parameter related to this isotherm is *n*_F_, which is the heterogeneity factor, when *n*_F_ is ranged from 1 to 10, the adsorption process is favoured, this trend was accomplished for both adsorbents (Table 3).

The lowest R^2^ values (0.942 for γ–CDs–EPI and 0.977 for HP–γ–CDs–EPI) were observed by adjusting the experimental data to the Langmuir isotherm. For this isotherm, it is interesting to analyse the value of *q*_max_ (maximum adsorption for the adsorbent under specific experimental conditions), both polymers showed similar ability to entrap the dye (11.92 mg/g for γ–CDs–EPI and 14.60 mg/g for HP–γ–CDs–EPI). Table 4 shows an exhaustive comparison and analysis of different *q*_max_ values for different CDs adsorbents and dyes. Apart from *q*_max_, the *R*_L_ parameter must be considered for this isotherm. It is a dimensionless constant and is described by the following Equation (13):

(13) $$R_{L}=\frac{1}{1+a_{L}C_{o}}\;\;$$

Finally, the experimental data were adjusted to the Temkin isotherm. The best adjustment for γ–CDs–EPI was achieved by using this model (0.946). This could be attributed to the adsorption happening onto heterogeneous surfaces [37]. The Temkin model demonstrated that the heat of adsorption decreased during the adsorption process [38]. The results for the *b*_T_ parameter were 1.053 kJ/mol and 0.890 kJ/mol for γ– and HP–γ–CDs–EPI polymers, according to the range of binding energies stated on the literature, these results suggested that physicochemical forces were influencing the adsorption of DR on to the CD adsorbents [39].

When the results obtained for the separation factor ranges from 0 to 1, the adsorption is a favourable process and, according to our results, in both adsorbents the *R*_L_ value was within this range, indicating and confirming that the adsorption process was favorable for both polymers as it was showed by the *n*_F_ parameter of Freundlich isotherm.

Gibbs free energy value (Δ*G*°) indicates the spontaneity of the process being an essential tool to predict the development of chemical reactions. To calculate this value, the equation employed was:

(14) $$K{}^{\circ}=\;K_{p}\times M_{\mathrm{adsorbate}}\times 55.5$$

K_p_ is the equilibrium constant (L/g), *M*_adsorbate_ is the MW of DR and 55.5 is the constant related to the mole concentration of water (mol/L) [54,55]. The result obtained from Equation (14) was used in Equation (15) to elucidate the spontaneity of this adsorption process.

(15) ΔG°= −RTlnK°

According to the results observed in Table 3, the standard free energy (Δ*G*°) was −25,175.97 for γ–CDs-EPI and −24,691.24 J/mol for HP–γ–CDs–EPI at 25 °C. The exergonic values obtained indicated that the adsorption process is spontaneous at the temperature tested.

The main advantages of the new synthesized polymers containing CDs are listed in Table 5.

### 3.5. Advanced Oxidation Process (AOP)

**Figure 10:**
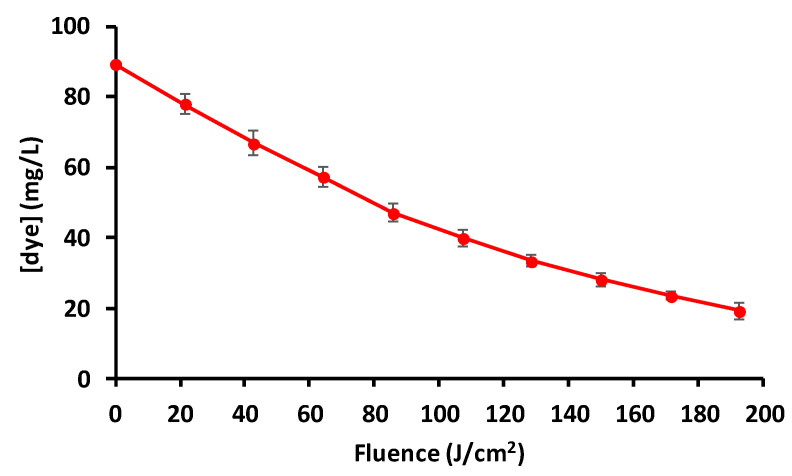
Decolourization of Direct Red 83:1 by an advanced oxidation process based on hydrogen peroxide and pulsed light. Bars mean standard deviations (*n* = 3).

The AOP was able to eliminate 78% of the dye from the solution after application of a PL treatment regime of 193 J/cm^2^ (Figure 10). The treatment involved the application of 90 light pulses. Taking into account that some common PL systems available in the market work with a pulse repetition rate of three pulses per second [56], the treatment could achieve that level of dye degradation in just 30 s, which makes it very fast. The pseudo-first kinetic constant of this process was 0.0079 ± 0.0004 cm^2^/J. The CD adsorption process followed by the AOP was able to eliminate more than 90% of the dye from the solution.

## 4. Conclusions

The synthesis of two new cyclodextrin adsorbents was achieved successfully in order to remove DR 83:1 from wastewater. γ– and HP–γ–CDs–EPI showed similar behaviour in the adsorption process. The adjustment of the experimental results to PFOM, PSOM and IDM indicated that in both cases the results were well fitted by using the PSOM, indicating that the adsorption depends on chemical forces, apart from the fact that the IDM plays a key role in the process, showing that in the adsorption of DR both chemical and physical forces are involved.

According to the determination coefficient, γ–CDs–EPI followed the Temkin isotherm, whereas in the case of HP–γ–CDs–EPI, the Freundlich isotherm presented the best adjustment to the experimental data. The maximum adsorption ability of both adsorbents (*q*_max_) was similar. In order to remove the residual concentration of DR in water, an AOP was considered after using the CDs polymers. More than 95% of DR was removed from water by combining CDs polymers and the AOP at the highest concentrations of dye.

## Figures and Tables

**Table 1:** Properties of both polymeric adsorbents.

Adsorbent Properties	γ–CDs–EPI	HP–γ–CDs–EPI
Swelling capacity (Qeq, g/g)	0.59 ± 0.02	0.61 ± 0.03
Porosity (%)	55.6 ± 2.5	52.1 ± 1.1
Density (g/cm^3^)	1.4 ± 0.1	1.5 ± 0.2
Particle size distribution D[4:3] (μm)	54	555
Span values	6.7	1.8

**Table 2:** Kinetics results for the three models analysed.

Pseudo-First Order Model (PFOM)								
	**γ–CDs–EPI**				**HP–γ–CDs–EPI**			
***C*_o_ (mg/L)**	***q*_e exp_**	***q*_e cal_**	**k_1_ (min ^−1^)**	**R^2^**	***q*_e exp_**	***q*_e cal_**	**k_1_ (min ^−1^)**	**R^2^**
**25**	1.126	0.597	0.065	0.853	1.146	0.517	0.065	0.817
**50**	2.259	1.520	0.065	0.935	2.213	1.238	0.065	0.895
**75**	3.444	2.421	0.058	0.941	3.352	2.275	0.064	0.944
**100**	4.503	3.589	0.086	0.980	4.408	2.426	0.080	0.905
**150**	5.924	5.035	0.034	0.962	6.129	4.581	0.061	0.958
**200**	7.315	6.223	0.029	0.947	8.076	6.591	0.048	0.973
**250**	8.821	7.585	0.025	0.905	9.667	6.295	0.045	0.890
**300**	10.690	9.462	0.022	0.909	10.985	9.268	0.056	0.983
**Pseudo-Second Order Model (PSOM)**								
***C*_o_ (mg/L)**	***q*_e exp_**	***q*_e cal_**	**k_2_ (g/mg min)**	**R^2^**	***q*_e exp_**	***q*_e cal_**	**k_2_ (g/mg min)**	**R^2^**
**25**	1.126	1.148	0.293	0.998	1.146	1.160	0.416	0.999
**50**	2.259	2.341	0.104	0.999	2.213	2.272	0.147	0.999
**75**	3.444	3.623	0.052	0.999	3.352	3.484	0.068	0.999
**100**	4.503	4.672	0.059	0.999	4.408	4.504	0.110	0.999
**150**	5.924	6.849	0.0079	0.996	6.129	6.410	0.028	0.999
**200**	7.315	8.771	0.0049	0.995	8.076	8.695	0.012	0.998
**250**	8.821	10.869	0.0030	0.983	9.667	10.309	0.014	0.999
**300**	10.690	14.492	0.0016	0.966	10.985	11.764	0.011	0.998
**Intraparticle Diffusion Model (IDM)**								
***C*_o_ (mg/L)**	***q*_e exp_**	**(*C*)**	**k_i_ (mg/g min^1/2^)**	**R^2^**	***q*_e exp_**	***q*_e cal_ (*C*)**	**k_i_ (mg/g min^1/2^)**	**R^2^**
**25**	1.126	0.879	0.0253	0.622	1.146	0.959	0.019	0.667
**50**	2.259	1.569	0.0738	0.701	2.213	1.719	0.052	0.748
**75**	3.444	2.205	0.133	0.747	3.352	2.329	0.109	0.737
**100**	4.503	3.153	0.148	0.575	4.408	3.611	0.086	0.649
**150**	5.924	1.735	0.417	0.931	6.129	3.831	0.243	0.729
**200**	7.315	1.568	0.573	0.952	8.076	3.958	0.421	0.832
**250**	8.821	1.302	0.719	0.959	9.667	5.897	0.393	0.865
**300**	10.690	0.458	0.993	0.960	10.985	5.818	0.551	0.761

**Table 3:** Adsorption isotherm constants.

Isotherm	Parameters	γ–CDs–EPI	HP–γ–CDs–EPI
Freundlich	*K*_F_ (mg/g) (L/mg) ^1/n^	0.992	0.818
	*n*_F_	1.893	1.626
	*R* ^2^	0.920	0.982
Langmuir	*q*_max_ (mg/g)	11.92	14.60
	*K*_L_ (L/g)	0.541	0.498
	*a*_L_ (L/mg)	0.045	0.034
	Δ*G* (J/mol)	−25175.97	−24691.24
	R^2^	0.942	0.977
	*R*_L_	0.468–0.068	0.539–0.089
Temkin	*a*_T_ (L/g)	0.604	0.480
	*b*_T_ (J/mol)	1.053	0.890
	R^2^	0.946	0.955

**Table 4:** Efficiency of different CDs crosslinked polymers in the removal of dyes.

Polymer	Dye	*q*_max_ (mg/g)	Experimental Conditions	Reference
MNP–β–CDs–GO	Malachite green	740	Adsorbent: 5 mg Dye: 50–700 mg/L 25–45 °C	[40]
β–CDs–PVA	Indigo carmine	287–495	Adsorbent: 0.01g Dye: 90–720 mg/L 25 °C	[41]
β–CDs–MNP	Rhodamine B Methylene Blue	250 333	Adsorbent: 0.5–2 g/L Dye: 50–500 mg/L 25 °C	[42]
CM–β–CDs–MNP	Methylene Blue	140–277	Adsorbent: 120–130 mg Dye: 0.1–3 mg/mL 25 °C	[43]
HNT–CDs	Rhodamine B	226	Adsorbent: 5 mg Dye: 2 × 10^−5^ M 25 °C	[44]
β–CDs–EPI	DR 83:1	107.5	Adsorbent: 1 g Dye: 25–300 mg/L 25 °C	[17]
β–CDs–CA	Methylene Blue	105	Adsorbent: 0.1 g Dye: 10–50 mg/L 30 °C	[45]
β–CDs–EPI	Malachite green	91.9	Adsorbent: 0.15 g Dye: 20–200 mg/L 25 °C	[46]
β–CDs–PE	p-Nitrophenol Phenolphthalein Naphthenates	20–75	Adsorbent: 20 mg Dye: 10–100 mg/L 25 °C	[47]
β–CDs–MIP	Trichromatic mixture	35.0	Adsorbent: 0.05 g Dye: 10–70 mg/L 25 °C	[48]
α–CDs–EPI	DR 83:1	31.5	Adsorbent: 1 g Dye: 25–300 mg/L 25 °C	[16]
HP–α–CDs–EPI	DR 83:1	23.4	Adsorbent: 1 g Dye: 25–300 mg/L 25 °C	[16]
β–CDs–TFT	Bisphenol A	19–24	Adsorbent: 0.5 mg/mL Dye: 0.04 mM 25 °C	[49]
β–CDs–EPI	Direct Blue 78	23.5	Adsorbent: 1 g Dye: 25–300 mg/L 25 °C	[7]
HP–β–CDs–EPI	DR 83:1	18.2	Adsorbent: 1 g Dye: 25–300 mg/L 25 °C	[17]
HP–γ–CDs–EPI	DR 83:1	14.6	Adsorbent: 1 g Dye: 25–300 mg/L 25 °C	This work
γ–CDs–EPI	Direct Blue 78	14.15	Adsorbent: 1 g Dye: 11–70 mg/L 25 °C	[50]
γ–CDs–EPI	DR 83:1	11.9	Adsorbent: 1 g Dye: 25–300 mg/L 25 °C	This work
β–CDs–HDMI	Evans Blue Chicago Sky Blue	10.6–12.1	Adsorbent: 25 mg Dye: 1 × 10^−3^ M 25 °C	[51]
β–CDs–MDI	Evans Blue Chicago Sky Blue	7.6–9.6	Adsorbent: 25 mg Dye: 1 × 10^−3^ M 25 °C	[51]
β–CDs–EPI	p-Nitrophenol	0.8	Adsorbent: 20 mg Dye: 0.2–10 mM 22–45 °C	[52]
β–CDs–EDTA	Methylene Blue Safranin O Crystal Violet	0.26 0.17 0.28	Adsorbent: 10 mg Dye: 10–500 mg/L 25 °C	[53]

EPI: epichlorohydrin, MIP: molecular imprinted polymers, TFT: tetrafluoroterephthalonitrile, MDI: 4,4′-methylene-bis-phenyldiisocyanate, HDMI: hexamethylenediisocyanate, EDTA: ethylenediaminetetraacetic acid, MNP: magnetic nanoparticles, HNT: halloysite, CA: citric acid, GO: graphene oxide, PE: polyurethane, PVA: polyvinyl alcohol.

**Table 5:** Advantages of CDs polymers.

Advantages of CDs Polymers
Limited use of reagents employed in the synthesis
Cyclodextrins are cheap compounds
No toxic or harmful components remains in the polymeric adsorbents
Cyclodextrin polymers can be stored for long periods
These polymers can be reused many times effectively
The desorption is easily achieved

## Supplementary Materials

The following are available online at https://www.mdpi.com/2073-4360/12/9/1880/s1, Figure S1: 1H NMR spectra of γ–CD, HP–γ–CD, γ– and HP–γ–CD EPI polymers.
