# Supplementary material for: Synthesis of New Cyclodextrin-Based Adsorbents to Remove Direct Red 83:1
Source: Polymers (Basel). 2020 Aug 20;12(9):1880. doi: 10.3390/polym12091880 (PMC7576497; doi:10.3390/polym12091880)
Supplement: Supplementary file 1 [file polymers-12-01880-s001.pdf]

SUPPLEMENTARY MATERIAL

Synthesis of new cyclodextrin-based adsorbents to remove Direct Red 83:1

José A. Pellicer, María Isabel Rodríguez-López, María Isabel Fortea, Vicente M. Gómez-López, David Auñó, Estrella Núñez-Delicado, and José A. Gabaldón *

Dpto. de Ciencias de la Salud. Universidad Católica San Antonio de Murcia (UCAM), Avenida de los Jerónimos 135, 30107 Guadalupe, Murcia, Spain.

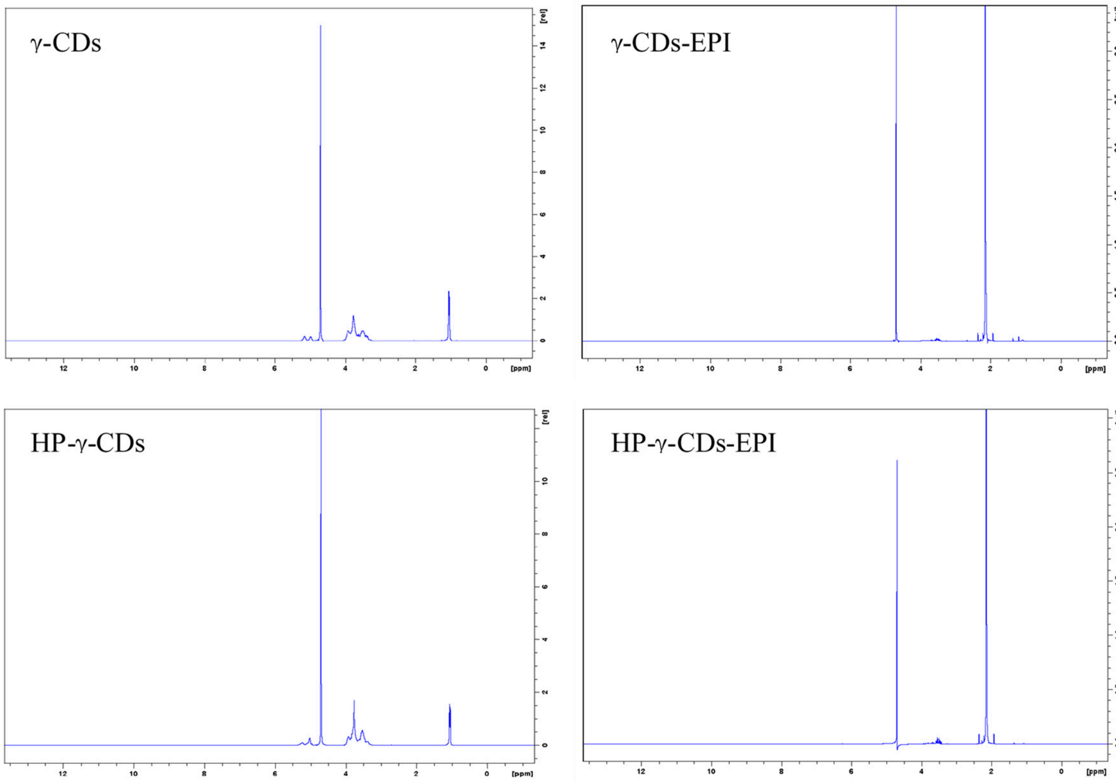

Fig. S1. ^{1}H NMR spectra of γ–CD, HP–γ–CD, γ– and HP–γ–CD EPI polymers.
